# Posthepatectomy liver failure

**DOI:** 10.3906/sag-2006-31

**Published:** 2020-10-22

**Authors:** İlhan OCAK, Serdar TOPALOĞLU, Koray ACARLI

**Affiliations:** 1 Department of Critical Care Unit, İstanbul Memorial Hospital, İstanbul Turkey; 2 Department of Surgery, School of Medicine, Karadeniz Technical University, Trabzon Turkey; 3 Department of Organ Transplantation, Department of Surgery, İstanbul Memorial Hospital, İstanbul Turkey

**Keywords:** Posthepatectomy liver failure, liver surgery, prevention, treatment

## Abstract

Liver surgery is one of the most complex surgical interventions with high risk and potential for complications. Posthepatectomy liver failure (PHLF) is a serious complication of liver surgery that occurs in about 10% of patients undergoing major liver surgery. It is the main source of morbidity and mortality. Appropriate surgical techniques and intensive care management are important in preventing PHLF. Early start of the liver support systems is very important for the PHLF patient to recover, survive, or be ready for a liver transplant. Nonbiological and biological liver support systems should be used in PHLF to prepare for treatment or organ transplantation. The definition of the state, underlying pathophysiology and treatment strategies will be reviewed here.

## 1. Introduction

Liver surgery is one of the most complex surgical interventions with high risk and potential for complications. Postohepatectomy liver failure (PHLF) is the main source of morbidity and mortality after major liver surgery. Despite major improvements in results after major liver surgery due to improvements in the maintenance and operation technique in the intensive care units, PHLF remains one of the most serious complications of major liver surgery [1,2]. Although a lower rate of PHLF has been reported in many studies in East Asian countries (1–2%); PHLF remains an important source of morbidity and mortality.

## 2. Definition

Although the definition of PHLF varies greatly between countries and groups, in 2011, the International Study Group of Liver Surgery (ISGLS) proposed a standardized definition and rating of PHLF after evaluating more than 50 studies. According to ISGLS; the deterioration (increase in INR and bilirubin levels) in the synthesis, excretion, and detoxification functions of the liver after liver surgery (day 5 or later) was defined as PHLF [1]. The incidence is reported at around 10% [1,2]. The highest acceptable mortality rate for major liver resection (LR) is considered as <10% and PHLF is shown as the most important (between 60% and 100%) cause of mortality [3]. Twenty-five percent of patients who die due to PHLF are lost after the first month postoperatively [4]. 

## 3. Pathophysiology

Hepatocytes and nonparenchymal cells must be present in adequate numbers for healthy regeneration of the liver remnant. The patency of inflow and outflow of remnant liver is another important factor for regeneration. In addition, factors that promote ongoing parenchymal damage after LR, notably small for size syndrome (SFSS), sepsis, and ischemia-reperfusion (IR) injury must be absent [5,6]. Hyperperfusion theory is the most widely accepted explanation of SFSS. Reduction in parenchymal volume and constant blood flow lead to a cycle of sinusoidal dilatation, shear stress, hemorrhagic infiltration, centrilobular necrosis, prolonged cholestasis, impaired synthetic function, and inhibition of cell proliferation [7,8].

The population of Kupffer cells is reduced after LR. Therefore, immune response is impaired and susceptibility to infection is increased. A relative increase in endotoxin delivery to the liver remnant is beneficial as it leads to activation of Kupffer cells and initiation of regeneration [9]. However, prolonged endotoxemia in sepsis leads to Kupffer cell dysfunction, impaired liver regeneration, and hepatic necrosis [10,11].

After the induction of ischemia, the complement cascade is activated, leading to Kupffer cell activation, generation of reactive oxygen species, and endothelial cell damage. In the reperfusion period, a cycle of cell adhesion molecule upregulation, cytokine release, T cell and polymorphonuclear cell recruitment and activation are initiated. Finally, microvascular injury, Kupffer cell-mediated inflammation, and hepatocyte death occur [12,13]. 

## 4. Risk and prevention

Risk factors for PHLF are summarized in Table 1. PHLF is divided into three subgroups according to the classification made by ISGLS (Table 2) [1]. Patients in group A with temporary liver dysfunction that do not require invasive treatment should be monitored. Group B and C patients with multiple organ failure or severe liver failure should be monitored under intensive care conditions. Patients should be closely monitored for signs of systemic inflammatory response syndrome (SIRS). Serum bilirubin, aminotransferase, albumin, international normalized ratio (INR), ammonia, lactate, and, C-reactive protein (CRP) levels should be closely monitored with serial measurements. Also, it is recommended that the patient group, whose antithrombin-3 activity measures below 61.5% on the first postoperative day, should be carefully monitored for failure [14]. Whether there is a problem with arterial, portal venous blood supply or venous outflow (hepatic veins) of the liver in patients who develop PHLF should be evaluated by Doppler ultrasonography, computerized tomography (CT), or angiography. In the presence of arterial stenosis-congestion, tissue plasminogen activator (t-PA) infusion or balloon angioplasty can be applied to the relevant area; the factors that reduce or stop the flow of the artery of interest should be eliminated with relaparotomy, and if necessary, reanastomosis should be performed [15]. In the presence of portal vein stenosis or thrombosis, systemic heparinization should be initiated with caution. In case of stenosis or bending, additionally, t-PA infusion can be tried by percutaneous entry into the portal vein and this stenosis or bending can be corrected by the endovascular stent in the early period. The obstructive jaundice condition in the postoperative period that may occur after surgery should also be examined, and the treatment process should be carefully managed in case of its presence (percutaneous drainage or relaparotomy is planned according to the patient’s condition) [16]. Strategies for prevention of PHLF are summarized in Table 3 [17–19]. 

**Table 1:** Risk factors for PHLF.

Patient-dependent factors	- Diabetes mellitus- Obesity- Liver damage due to chemotherapy- Malnutrition- Kidney failure- Hiperbilirubinemia- Thrombocytopenia- Lung disease- Cirrhosis/chronic liver disease- Age > 65
Surgery-dependent factors	- Bleeding during surgery > 1200 mL- Massive transfusion in surgery- Vascular resection requirement->50% resection of liver volume- Major hepatectomy including right lobe- Excessive dissection of the hepatoduodenal ligament- Remnant liver volume <25% - Operating time > 240 min.- Prolonged application of Pringle or TVE maneuver
Postoperative factors	-Postoperative bleeding -İntraabdominal infection

**Table 2:** PHLF classification by ISGLS (International Study Group of Liver Surgery).

Group	Clinical description	Diagnosis	Symptoms	Mortality
A	Impaired liver function	-Urine Output >0.5 mL/kg/h-BUN <150 mg/dL-Oxygen saturation >90%-INR <1.5	None	%0
B	Deviation from the expected postoperative course, no need for invasive support	-Urine Output ≤0.5 mL/kg/h-BUN <150 mg/dL- Despite the oxygen supply oxygen saturation <90%-INR ≥1.5, <2.0	-Acid-Weight gain-Mild respiratory failure-Confusion-Encephalopathy	%12
C	Multiple organ failure requiring invasive support	-Urine output ≤0.5 mL/kg/h-BUN ≥150 mg/dL- Despite high fractionated oxygen support oxygen saturation ≤85%-INR ≥2.0	- Kidney failure- Hemodynamic instability- Respiratory failure- Massive ascites-Encephalopathy	%54

PHLF: posthepatectomy liver failure; BUN: blood urea nitrogen; INR: international normalized ratio.

**Table 3:** Strategies for prevention from PHLF.

Safe surgery group incirrhotic patients	-Child-Pugh Group A patients- Platelets >100,000/mL- No clinically apparent portal hypertension- Liver volume remaining between > 40% and 50%- Indocyanine green retention <15%
Preoperative strategies	- Increasing the liver volume left behind by portal vein embolization- Ensuring overweight patients to lose weight before surgery- Nutritional support- Control of diseases that will cause additional morbidity- Preoperative measurement of liver stiffness by transient elastography- Preoperative measurement of spleen thickness
Intraoperativestrategies	- Avoiding unnecessary dissection of the hepatoduodenal ligament, and if necessary, carefully dissecting- Minimizing blood loss by performing parenchymal resection under low central venous pressure- Ischemic preparation- Application of intermittent Pringle maneuver- Hypothermic liver protection- Surgery combined with ablation treatments- Two-stage resection- Avoiding blood transfusion as much as possible- Compliance with the principles of hemostasis
Postoperative strategies	- Early detection and treatment of postoperative bleeding- Early detection and treatment of postoperative bile duct obstruction or bile leak- Early detection and treatment of postoperative intra-abdominal infection

PHLF: posthepatectomy liver failure.

## 5. Management and treatment

### 5.1. Medical support therapy

The approach to patients with PHLF starts with medical support therapy. When SIRS is observed in patients, hypotension and relative hypovolemia, observed due to decreased systemic vascular resistance should be monitored by invasive monitoring. Colloid-weighted fluids should be used in fluid replacement, and albumin support should be provided. Vasoactive agents may be required in cases that do not respond despite adequate volume support. Extracellular fluid accumulation should be avoided [20]. Hydrocortisone support is recommended for the control of persistent lactic acidosis caused by hypoperfusion and vasopressor agent use. N-acetyl cysteine should also be administered in the treatment of liver failure [21,22]. Proton pump inhibitor therapy should be applied to prevent the development of stress ulcers. Early intubation and mechanical ventilator therapy may be needed since patients with liver failure may develop acute lung injury (PaO2 / FiO2 ratio < 300 mmHg) or acute respiratory distress syndrome (PaO2 / FiO2 ratio < 200 mmHg). Tidal volume should be 6 mL/kg in adult ventilator therapy and PaO2 should be kept above 80 mmHg. Also, high positive end-expiratory pressure (PEEP) administration should not be applied at high levels as it will cause hepatic congestion, portal hypertension, acid development, and decreased liver regeneration. Hyperventilation (PCO2; 25–30 mmHg) protocol should be applied to decrease the intracranial pressure in patients who need mechanical ventilator treatment. The most important underlying cause of the encephalopathy in the liver failure is ammonia accumulation and cerebral edema due to hyponatremia. Since brainstem herniation or hypoxic brain injury are complications that may develop due to brain edema and cause a rapid deterioration of the patient, treatment preventing the formation of brain edema should be started (mannitol therapy, hyperventilation, sodium thiopentone, hypertonic fluid therapy, etc.). [20,23]. Treatment using oral rifaximin, laxative (lactulose), and enema limits the formation of ammonia. In patients with grade 3–4 encephalopathy, monitoring intracranial pressure, close blood sugar monitoring, and controlled hypothermia are recommended [3]. The development of resistant hypoglycemia (disruption of hepatic gluconeogenesis and hyperinsulinemia) is a poor prognostic marker. First, enteral nutrition should be applied and parenteral nutrition should be given to patients with limited oral intake. The daily calorie need of patients should be calculated between 25 and 35 kcal/kg and daily protein support between 1 and 1.2 g/kg. Branched-chain amino acid solutions (leucine, isoleucine, or valine) should be preferred to meet protein needs. Most of the calorie needs should be met with carbohydrate and fat solutions. Acute tubular necrosis due to SIRS or development of hepato-renal syndrome (HRS) due to underlying liver disease should be monitored and treatment of complications such as hypokalemia (resistant to diuretic therapy), hypophosphatemia oliguria, hyponatremia, and water retention should be carried out immediately [20,21]. Massive ascites is particularly observed in patients with preoperative portal hypertension. Furosemide/spironolactone should be administered at a rate of 2/5 (20 mg / 50 mg) in diuretic treatment. The diuretic response may be limited due to acute renal injury due to surgery, SIRS, or HRS. Also, diuretic use deepens the existing hyponatremia. When sodium levels fall below 120 mEq/L, diuretic therapy should be discontinued and patients with intravascular volume deficits should be given albumin support. Intermittent paracentesis should be performed in case of impaired patient comfort, restricted breathing, impaired oral intake, or leakage of ascites from the surgical area (in patients with liver failure). In the case of paracentesis more than 5 L, 8 g of albumin replacement should be performed for each liter taken to prevent renal failure, hyponatremia, and hypotension. TIPS or peritoneovenous shunt may be required in the presence of prolonged acid (4–6 weeks and above postoperatively in liver failure patients). Bacterial infections (80%) are found in the majority of patients with liver failure. Although prophylactic antibiotic therapy is not recommended, it is recommended to start broad-spectrum antibiotic therapy without waiting for culture results in the presence of the smallest suspicion [20,21]. It is also recommended to add antifungal drugs to treatment. 

Factor II, VII, IX, and X dysfunction occurs depending on the decarboxylation of the degraded vitamin K in the liver failure. Also, disorders of thrombocytopenia and thrombocyte function are observed due to renal dysfunction and uremia. Fresh frozen plasma (FFP) is used to control oncotic pressure and prevent INR rise. However, large amounts of FFP transfusions should be used with caution as they can lead to the development of brain edema and acute lung injury. The risk of bleeding should be taken into account during deep vein thrombosis prophylaxis application to patients. 

### 5.2. Liver support systems in treatment

These systems are developed to support patients with liver failure until the patients’ condition improves or a transplant is made. The complex physiological, biochemical, and metabolic functions of the liver do not make it possible to perform a truly complete replacement therapy. Also, the complexity of the pathophysiology of liver failure, especially the inability to reveal the underlying mechanisms affecting prognosis, such as cerebral edema and encephalopathy, is an important barrier to supportive therapies. Approaches to liver support are divided into two groups as nonbiological and biological systems. Nonbiological systems are based on nonspecific detoxification using a limited permeable membrane. Biological support systems try to create a detoxification environment close to natural liver tissue by utilizing various cell (hepatocyte) cultures [20,21,24]. Nonbiological support units are used in most of the European countries and our center due to the high cost of biological systems, technical difficulties in supplying hepatocytes and maintaining their viability for a long time. 

### 5.2.1. Nonbiological liver support

Nonbiological support units are applied with extracorporeal pump machines with different features. Generally, there are options on the used pump machines that allow different support units to be applied. Applications are made through 2-way wide lumen catheters placed in the subclavian, internal jugular, or femoral vein. The main purpose in these applications is to remove the molecules other than essential hormones, growth factors, immunoglobulins, coagulation factors, and complement system proteins (molecular weight > 50–60 kDa), which are bound to carrier proteins, from circulation. In this way, water-soluble toxins (ammonia, urea, lactate, creatinine, etc.) and oil-soluble toxins (bile acids, bilirubin, aromatic amino acids, short and medium-chain fatty acids, etc.) can be effectively removed. Also, tumor necrosis factor (TNF)-α (17.5 kDa), Interleukin (IL)-1β (17 kDa), IL-6 (21 kDa), IL-8 (8 kDa) and IL-10 (18.7 kDa) are among the main cytokines that play an active role in the etiopathogenesis of liver failure and removing them from circulation also aims to correct the clinical picture of the patients. [25,26].

**Figure 1:**
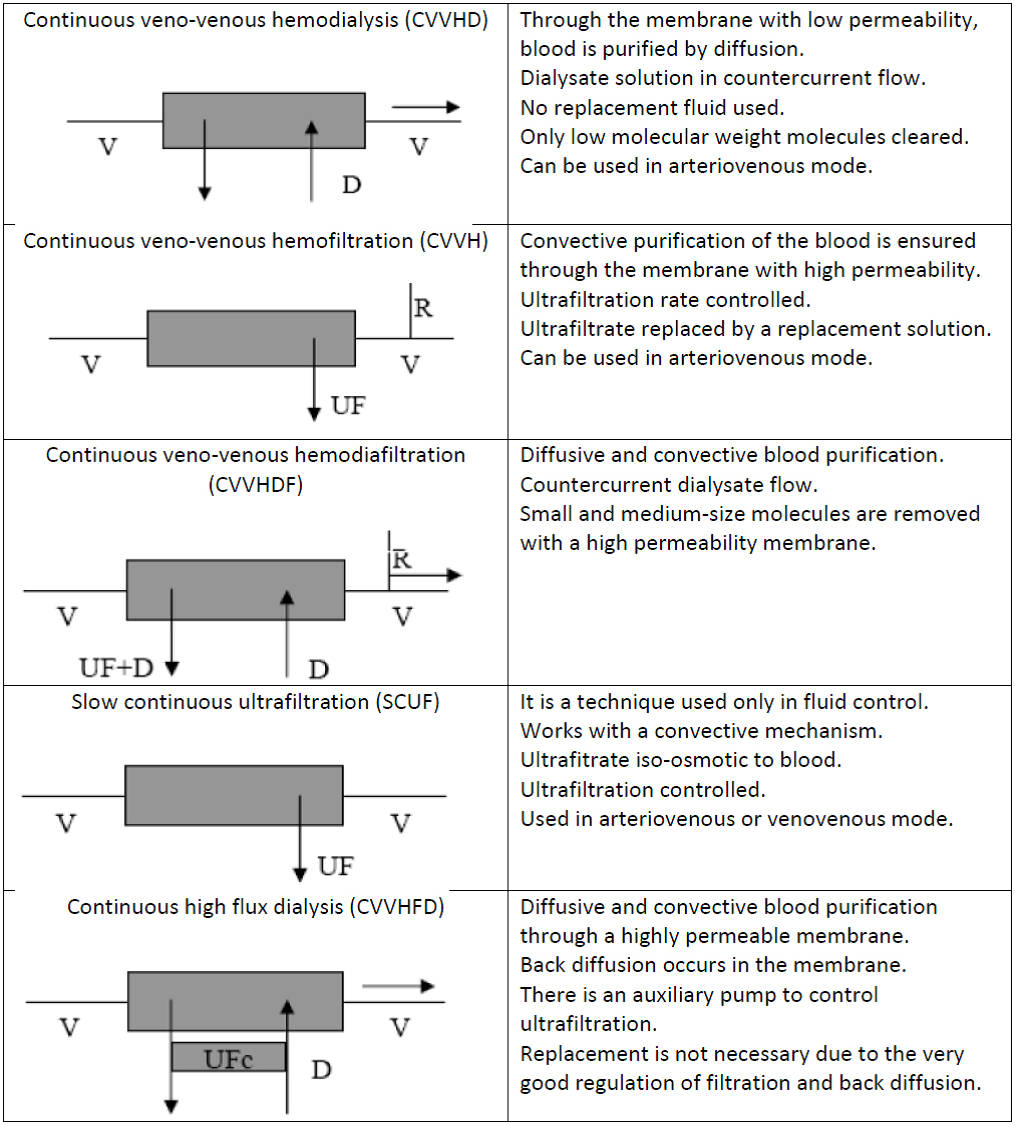
Continuous types of renal replacement therapy and basic working mechanisms. V: vein; D: dialysate; R: replacement solution; UF: ultrafiltrate; UFc: ultrafiltrate control pump.

Nonbiological liver support systems are divided into 4 main groups [27];

I. Continuous renal replacement therapies (CRRT); continuous venovenous hemodialysis, continuous venovenous hemofiltration (CVVF), continuous venovenous hemodiafiltration (CVVHDF), continuous slow ultrafiltration, and continuous high-flux dialysis (Figure 1)

II. Plasmapheresis, plasma exchange, and continuous plasma filtration adsorption

III. Hemoperfusion

IV. Albumin dialysis 

## 5.2.1.1. Continuous renal replacement therapies (CRRT)

Although it is usually done by a large lumen central catheter, it can also be done using arteriovenous (AV) fistula. Venous blood from the patient enters the peristaltic pump with a venovenous circuit. According to the intermittent hemodialysis application, it is aimed to lessen the patient’s hemodynamics by drawing fluid continuously in a limited volume [26]. During the cycle, coagulation is prevented using citrate or heparin. CRRT is mainly used to extract excess fluid in the extracellular space. They are used effectively in removing toxins that are not bound to albumin [27,28]. The membranes used in the units are made of biocompatible material (polyacrylonitrile, polymethylmethacrylate, etc.) to limit the activation of complement and other humoral systems. The tendency towards coagulation is minimal due to the high ultrafiltration constant. Dialysate and replacement fluid are used during the procedure with the selected CRRT technique. Dialysate is the liquid in which toxins and waste materials collected from the blood exist. The replacement fluid is a balanced electrolyte solution added to the venous blood which returns to the patient to maintain body homeostasis before or after the filter through which the blood passes. It is aimed to maintain the normal electrolyte and acid-base state while forming the composition. The sodium concentration in the liquids used is 150 mmol/L. If necessary, KCl, calcium, and magnesium can be added. The pH can be buffered using bicarbonate or lactate. Although heparin (nonfractionated) is often preferred for anticoagulation of the system, low molecular weight heparin, citrate, prostacyclin, or nafamostatmesylate can also be used [26,29]. After the procedure, the blood is given to the patient again with the replacement fluid or without replacement. Five different CRRTs can be made. Diffusion, convection, or a combination of both methods used in CRRT. The diffusion method is based on the exclusion of toxins dissolved in the blood. Toxins pass from one side of the semipermeable membrane (low permeability) to the other, depending on the electrochemical (concentration) gradient. The molecules move from the high concentration section to the low concentration section. Low molecular weight (5–15 kDa) toxins such as acid, potassium, and uremic toxins are discarded with this method but molecules reaching up to 30 kDa can be removed from the circulation with the use of synthetic polymeric membranes (polyacrylonitrile, polymethylmethacrylate, etc.). The convection method is based on ensuring the excretion of toxins dissolved in the blood. It works with a mechanism like the normal function of the human kidney. Solubles dissolve in the high-pressure zone with solvent and move from the high-pressure section to the low-pressure section through the high permeability membrane. In this mechanism, the transmembrane pressure gradient is important. Convection depends on filtration rate, membrane permeability, and soluble concentration. Medium-sized molecules (<60 kDa) are removed more effectively than in the diffusion method [23,24,26].

## 5.2.1.2. Plasmapheresis, plasma exchange, and continuous plasma filtration adsorption

**Figure 2:**
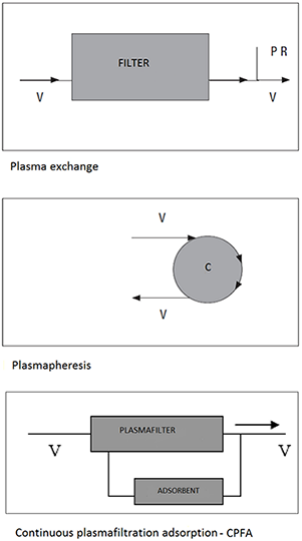
Working mechanisms of plasmapheresis and plasma exchange treatments. V: vein; PR: plasma replacement; C: centrifuge.

In plasma exchange application, while plasma which is separated from the blood of the patient with the help of high permeability membrane is taken out, the patient is given fresh frozen plasma and so the change is made. In the plasmapheresis method, plasma separated from the patient’s blood by centrifugation method is not replaced [27]. In continuous plasma filtration adsorption (CPFA) method, patient’s plasma is filtered with a high permeability plasma filter that allows it to pass through a bed of adsorbent material (carbon or resins) (Figure 2). Each treatment method, like the nonbiological liver support treatments mentioned earlier, is applied by central venous catheter and with module alteration of the same machines. The aim is to remove circulating antibodies and reduce cytokine load [29–31]. Large molecules ( >60 kDa) are removed using this method. Since these molecules include molecules such as growth hormones, immunoglobulins (150–900 kDa), albumin (66.3 kDa), transferrin (76 kDa), fibrinogen (341 kDa), the plasmapheresis method is especially used in many autoimmune diseases and ABO-incompatible or cross-match positive kidney transplantation. The plasma exchange method is used to remove bilirubin effectively from circulation, especially in cases of hyperbilirubinemia [32,33]. In the treatment of liver failure, plasma exchange, or plasmapheresis treatment together with CRRT is recommended [34–36]. In this way, it is aimed to ensure that the growth factors and hormones that remain useful for the patient remain in circulation. In a study published in Japan, CVVHDF and plasma exchange methods were used together in the treatment of acute liver failure. Patients’ consciousness improved with this treatment and brain edema and HRS did not develop during treatment [37]. In this study, the average number of sessions is 21 (4–30), 20% of patients with liver failure due to acute hepatitis B infection, and 57% of patients with liver failure due to an unknown cause. In the plasma exchange application, the sessions take 4 h and the plasma removed from the patient (40–50 mL/kg/session) is replaced with fresh frozen plasma (8–10 units/session) or Human albumin (5% H. Albumin, 2500 mL/session) or saline (3000 mL/session) [29,30]. 

## 5.2.1.3. Hemoperfusion

Hemoperfusion is the process of passing high blood volume (300 mL/min) of patient blood through an adsorbent surface especially to remove water-soluble toxins (ammonia, urea, lactate, creatinine, etc.) from the blood and give it back to the patient. The adsorbable chemical sorbents used in hemoperfusion are resin, activated carbon, or coal. Coal hemoperfusion is the most studied nonbiological liver supplement treatment. Initially, although it was observed that it was more effective than hemodialysis treatment in survival and improvement of the neurological picture in patients with liver failure; this difference could not be demonstrated in controlled studies. However, activated charcoal is still used in the most effective nonbiological liver support systems (MARS, PROMETHEUS) [38]. Resins (neutral, anionic, and cationic) separate substances that are protein-bound and cannot be removed by dialysis, such as bilirubin, bile acids, and barbiturates-nephrotoxic drugs from plasma. However, it causes hypotension, thrombocytopenia, leukopenia, and bleeding since it also holds clotting factors and other molecules [39]. The hemoperfusion method is applied in 4–5 h sessions, and the pump speed is adjusted to 160 mL/h during the procedure. 

## 5.2.1.4. Albumin dialysis (MARS and PROMETHEUS)

MARS (Molecular Adsorbent Recirculating System, Gambro AB, Stockholm, Sweden) or PROMETHEUS (Fractionated Plasma Separation, Adsorption, and Dialysis system, Fresenius Medical Care AG & Co. KGaA, Homburg, Germany) can be applied via the vascular route used in the treatment of continuous renal replacement. MARS consists of three main units. Continuous albumin dialysis circuit allows removal of protein-bound toxins (polysulfone membrane that allows passage of molecules smaller than 60 kDa that albumin cannot pass). The column that holds toxins bound to albumin reactivates albumin and ensures a return to circulation, thus preventing the support of albumin in large volumes. A continuous renal replacement circuit allows for classic hemofiltration or hemodialysis. The MARS cartridge needs to be replaced every 8 h [40–43]. In the PROMETHEUS system, the plasma of the patient containing albumin is separated by a membrane with a molecular permeability of 250 kDa and passed through two columns with different adsorbents. The substances dissolved in water are cleaned with a high exchange dialyzer. With both methods, the excretion of water-soluble metabolites such as ammonia, urea, creatinine, and albumin-bound substances such as bile acid and bilirubin is effective.

In nonbiological support units, heparinization is generally systemic, but rarely applied regionally (heparin infusion is initiated before the filter, and 10–20 mg/h protamine is given to the circulation after the filter). Heparin is given with a dose of 5–10 u/kg, ACT 200–250, and PTT are kept in the range of 1.5–2 times the normal value. Anticoagulation is not applied in patients with thrombocytopenia (<80,000/mL) or in plasmapheresis using isotonic NaCl solution as the clot is unlikely to form in the filter. With the application of citrate and anticoagulant, which have been used recently, complications related to heparin have also been eliminated. Citrate-anticoagulant application is included in the set. It is also neutralized with Calcium. Problems that may be encountered in applications with nonbiological support units are summarized in Table 4.

**Table 4:** Potential complications of nonbiological support system applications.

System	Complications
Cardiovascular system	Hypotension; hypovolemia, cardiac dysfunction or air embolismAngina, myocardial infarctionCardiac dysrhythmiasSteal syndrome; decrease of blood flow at distal to the vascular access
Respiratory system	Pulmonary alterations caused by hypoxemia, air embolism, leukocyte or complement induction
Neurological system	Disequilibrium syndrome; mental confusion, delirium, coma, seizureMuscle cramps
Hematological system	Bleeding, leukopenia, thrombocytopenia, hemolysis, DIC (mostly due to application of heparin)
Metabolic	Electrolyte and acid-base disorders
Dialysis problems	Dialysis rupture and clotting (occlusion)Dialysate contamination (fluoride)Mechanical complications
Problems with vascular access	Thrombosis and infection
Other	Allergic and bio-incompatibility reactions

DIC: disseminated intravascular coagulation.

## 5.2.1.5. Treatment algorithm

**Figure 3:**
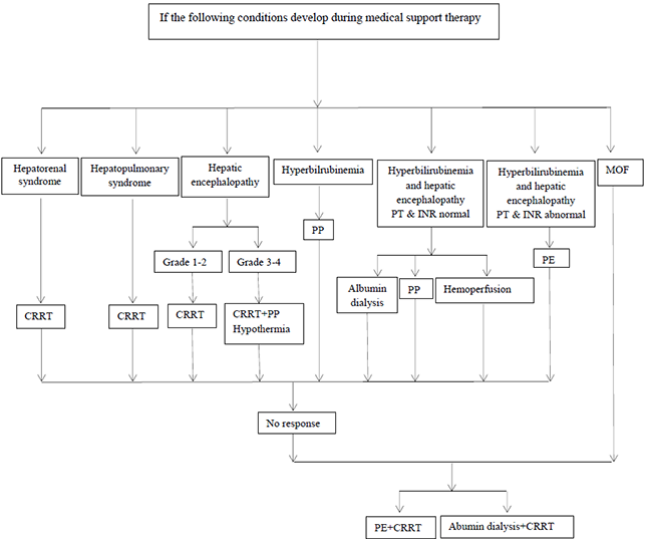
Flow diagram of nonbiological liver support systems in cases of liver failure. MOF: multiorgan failure; PT: prothrombin time; INR: international normalized ratio; PP: plasmapheresis; PE: plasmaexchange; CRRT; continuous renal replacement therapy; Albumin dialysis; MARS (Molecular Adsorbent Recirculating System) and PROMETHEUS (fractionated plasma separation, adsorption, and dialysis).

Nonbiological support units are activated in the presence of problems that arise or become more prominent during medical support treatment in the treatment of liver failure [44]. Although there is no consensus on which of the nonbiological support units should be started in patients who comply with the clinical parameters indicated in Figure 3, our preferred algorithm is summarized. When the occurrences that dominate the clinical course of liver failure (HRS, encephalopathy, hyperbilirubinemia, hepato-pulmonary syndrome, and multiorgan failure-MOF) are considered, treatments are shaped by emphasizing the different features of the support units. Treatment of CVVH or CVVHDF is preferred as the first option in the liver failure table dominated by HRS, plasma exchange, and CRRT or albumin dialysis and CRRT should be used together if there is no response. CVVH or CVVHDF is preferred in the occurrence of liver failure dominated by hepato-pulmonary syndrome, plasma exchange and CRRT or albumin dialysis and CRRT should be used together in unanswered cases. In liver failure, where mild hepatic encephalopathy is dominant, CVVH or CVVHDF is preferred as the first approach. In the presence of severe encephalopathy, plasmapheresis and controlled hypothermia are applied in addition to these treatments. In cases where there is no response to these treatments, plasma exchange and CRRT or albumin dialysis and CRRT should be used together. In the liver failure table where only hyperbilirubinemia is dominant, plasmapheresis treatment is started; plasma exchange and CRRT or albumin dialysis and CRRT should be used together if no response is obtained. Plasmapheresis, hemoperfusion, or albumin dialysis can be used as the first option in cases where hepatic encephalopathy is accompanied by hyperbilirubinemia and bleeding parameters are normal. In cases where there is no response, plasma exchange and CRRT or albumin dialysis and CRRT should be used together. Plasma exchange should be used as the first choice in cases where hyperbilirubinemia is accompanied by hepatic encephalopathy and bleeding parameters are impaired. In cases where there is no response, plasma exchange and CRRT or albumin dialysis and CRRT should be used together. Unlike other clinical pictures, plasma exchange and CRRT or albumin dialysis and CRRT should be used together in the MOF table.

When the transaction costs of nonbiological support units are examined, a fixed expense of around 5000 € is required per session in MARS or PROMETHEUS applications (the prices of extracorporeal pump machines are ignored). Fixed expenditure per session varies between 100 and 1000 € in CRRT [24]. The fixed expense is around 1000 € per session in plasma exchange applications. CRRT and plasma exchange applications come to the fore as more economical options in the creation of the treatment algorithm when considering the costs mentioned above.

## 5.2.2. Biological liver support systems

In a study conducted for the first time in 1956, urea was obtained from ammonium chloride using homogenate obtained from cow liver [45]. This study was followed by studies using the liver from many different animal species [38]. The complexity of the preparation process and the loss of effectiveness of the prepared homogenate in a short time made it difficult to adapt this approach to clinical use. The livers of different kinds of animals were used for perfusion (xenogenic extracorporeal liver perfusion) and the improvements in biochemical parameters and neurological signs were noted in a limited number of clinical studies [46,47]. The successful level achieved in hepatocyte isolation techniques paved the way for the use of hepatocytes in different configurations in liver support systems. The usage area of hepatocytes in liver failure can be summarized under two headings; implantation (hepatocyte transplantation) and extracorporeal systems. The beneficial effects of human hepatocyte transplantation in the treatment of liver failure have been demonstrated in a limited number of case reports [48]. However, there is no data on the use of xenogenic hepatocyte transplantation in the treatment of liver failure in humans. The most important obstacle to hepatocyte transplantation treatment is that toxic or viral factors leading to liver failure prevent the transplanted hepatocytes from organizing [38].

Extracorporeal systems or bioartificial liver support systems have been developed to perform detoxification by intermittently connecting to the human circulatory system, just like nonbiological systems. These systems consist of two main parts. The artificial unit consists of a bioreactor and parts of this reactor, while the other unit, the biological unit, consists of hepatocytes [38]. For the first time in 1987, Matsumura et al. placed isolated rabbit hepatocytes in the unit of the device separated from the patient’s circulation by cellulose membrane in a treatment they applied to a 45-year-old patient undergoing hepatic insufficiency due to inoperable biliary tract tumor [49]. Two years after this case report, Margulis et al. used the support unit with pig hepatocytes in their 126 patient series, providing a significant survival advantage, especially for patients before the coma [50]. Today, there are many bioartificial liver support systems developed by different study groups and used in clinical studies (Table 5). The human hepatocyte cell line (C3A) was used only in the ELAD (Extracorporeal Liver Assist Device) system among these systems. These cells have been cloned from the human hepatoblastoma cell line, their tumor-forming activities have been reduced and their albumin-alpha fetoprotein production activities have been increased [51]. In other systems, pig hepatocytes are used [52–56]. In Table 5, the cost of treatment of bioartificial liver support systems, which are briefly explained as working systems and treatment processes, is around 50,000–60,000 € and therefore not primarily preferred in our country and European countries [24].

**Table 5:** Major bioartificial liver support systems in clinical use.

	ELAD	HepatAssist	TECA-HALSS	BLSS	RFB	AMC-BAL
Study group	Houston	Los Angeles	Beijing	Pittsburgh	Cavezzo	Amsterdam
Cell type	Human	Pig	Pig	Pig	Pig	Pig
Cell source	C3A Culture	Cryopreserve	Freshisolation	Freshisolation	Freshisolation	Freshisolation
Cell quantity	200–400 g	5–7 × 109	10–20 × 109	70–120 g	200–230 g	10 × 109
Treatment duration	Max 168 h	6 h	Max 5 h	12 h	Max 24 h	Max 24 h
Anticoagulation	Heparin	Citrate	Heparin	Heparin	Heparin /Citrate	Heparin
Additional detoxification	None	Coal	Coal	None	None	None
Randomized controlled study	Yes	Yes	None	None	None	None

ELAD: extracorporeal liver assist device; TECA-HALSs: TECA-hybrid artificial liver support system; BLSS: bioartificial liver support system; RFB: radial flow bioreactor; AMC-BAL: AMC-bioartificial liver.

## 6. Conclusion

PHLF continues to be a serious surgical complication of the liver occurring in approximately 10% of patients undergoing major liver surgery. PHLF ranges from a mild hepatic impairment, characterized by transient hyperbilirubinemia, to hepatic impairment, which causes multiple system insufficiency that requires invasive treatment in the intensive care unit. Neoadjuvant therapy with obesity, diabetes, chemotherapy, underlying cirrhosis, increased age, male sex, extended liver resection need, and long-term operation with high intraoperative EBL (estimated blood loss) increases the risk of PHLF. Early start of the liver support systems is very important for the PHLF patient to recover, survive, or be ready for a liver transplant. The nonbiological and biological liver support systems described above should be used for treatment or organ transplant in PHLF, and the method of application should be with the joint approach of the Organ Transplant Clinic and Intensive Care Unit. The most effective treatment of liver failure is a liver transplant. However, since the organ pool is far from meeting expectations, both biological and nonbiological liver support systems should be expected to be used more and more effectively in the treatment of PHLF in the future.

**Conflict of interest**

The authors declare that they have no conflict of interest. This review was prepared without any support from funding agencies in the public, commercial, or nonprofit sectors.
